# Oligodendrogenesis and myelination tracing in a CRISPR/Cas9-engineered brain microphysiological system

**DOI:** 10.3389/fncel.2022.1094291

**Published:** 2023-01-19

**Authors:** July Carolina Romero, Cynthia Berlinicke, Sharon Chow, Yukan Duan, Yifei Wang, Xitiz Chamling, Lena Smirnova

**Affiliations:** ^1^Bloomberg School of Public Health, Center for Alternatives to Animal Testing, Johns Hopkins University, Baltimore, MD, United States; ^2^Department of Ophthalmology, Wilmer Eye Institute, Johns Hopkins University School of Medicine, Baltimore, MD, United States

**Keywords:** brain organoids, CRISPR/Cas9, hiPSC fusion KI line, neurodevelopment, oligodendrogenesis, myelination

## Abstract

**Introduction:**

Oligodendrocytes (OLs) are the myelin-forming cells of the central nervous system (CNS). Although OLs can be differentiated from human-induced pluripotent stem cells (hiPSCs), the *in vitro* modeling of axon myelination in human cells remains challenging. Brain microphysiological systems (bMPS, e.g. organoids) are complex three-dimensional (3D) cultures that offer an ideal system to study this process as OLs differentiate in a more *in vivo*-like environment; surrounded by neurons and astrocytes, which support the myelination of axons.

**Methods:**

Here, we take advantage of CRISPR/Cas9 technology to generate a hiPSC line in which proteolipid protein 1 (PLP1), an OLs marker, is tagged with super-fold GFP (sfGFP). While generating the PLP1-sfGFP reporter, we used reverse transfection and obtained higher Knock-In (KI) efficiency compared to forward transfection (61–72 vs. 46%).

**Results:**

After validation of the KI and quality control of the PLP1-sfGFP line, selected clones were differentiated into bMPS, and the fidelity, specificity, and function of the tagged PLP protein were verified in this model. We tracked different stages of oligodendrogenesis in the verified lines based on PLP1-sfGFP^+^ cells’ morphology, and the presence of PLP1-sfGFP surrounding axons during bMPS’ differentiation. Finally, we challenged the bMPS with cuprizone and quantified changes in both the percentage of PLP1-sfGFP expressing cells and the intensity of GFP expression.

**Discussion:**

This work demonstrates an efficient method for generating hiPSC KI lines and the description of a new 3D model to study OL differentiation, migration, and maturation both during *in vitro* neurodevelopment as well as in response to environmental chemicals or disease-associated stressors.

## 1. Introduction

Oligodendrocytes (OLs), the myelin-forming cells of the central nervous system (CNS), are very dynamic and they participate in axonal maintenance, performance, survival, and adaptation (Michalski and Kothary, 2015; Mohamed et al., 2021). They originate from oligodendrocyte precursor cells (OPCs) and play an important role in myelin repair in the adult brain (Gensert and Goldman, 1997). Following coordinated proliferation and migration, OPCs further differentiate to mature OLs, undergoing morphological changes and generating an extensive network of processes that establish contact with neighboring axons and ultimately wrap around these axons creating myelin sheaths (Michalski and Kothary, 2015). After myelin compaction (driven by myelin basic protein, MBP), OLs improve neural circuit performance and provide metabolic support to the wrapped axons. Importantly, several neurodevelopmental and neurodegenerative disorders have underlying altered myelination in the CNS (e.g., hypomyelinating leukodystrophies, Pelizaeus–Merzbacher disease, Allan–Herdon–Dudley syndrome, autism, multiple sclerosis, stroke, schizophrenia, autoimmune encephalomyelitis) stressing the prominent role of OLs in human health (Galvez-Contreras et al., 2020; Phan et al., 2020; Mohamed et al., 2021).

Most of the knowledge about OLs biology comes from rodent studies. However, since the timing of OPC/OL differentiation and maturation varies not only amongst species but also by the age of the organism, the anatomical location of the cell population, and injury response, rodent OL models have limitations when extrapolating to human OL biology (Jakovcevski et al., 2009; Bradl and Lassmann, 2010; Ornelas et al., 2016; Bribián et al., 2020). Thus, there is a need for human-relevant oligodendrogenesis and myelination models. As access to human CNS samples is limited, the development of human *in vitro* models that recapitulate key events of OL biology in their native microenvironment is essential. Advances in stem cell research have paved the road for the development of human microphysiological systems (MPS) such as 3D organoids and organs-on-chip, which more closely resemble human cell physiology compared to traditional monocultures or animal models (Alépée et al., 2014; Jensen and Teng, 2020; Marx et al., 2020; Hogberg and Smirnova, 2022). To this end, our lab and others have developed hiPSC-derived bMPS models with efficient and reproducible differentiation and maturation of neurons and supporting CNS cells (to name a few: Pamies et al., 2017a; Sloan et al., 2018; Albanese et al., 2020; Ahn et al., 2021). The bMPS differentiated using our protocol, consist of most of the neural cell types of the CNS (GABAergic, glutamatergic, dopaminergic neurons, neural progenitor cells (NPCs), astrocytes, and OLs), contain neurons with myelinated axons and are also electrically active (Pamies et al., 2017a; Chesnut et al., 2021; Modafferi et al., 2021; Huang et al., 2022). All together, these features make the model a great tool for advancing the current understanding of human brain development and disease in addition to its use for screening potential neurodevelopmental toxicants (Pamies et al., 2018a; Leite et al., 2019; Zhong et al., 2020).

Another revolutionary advance from the past decade that has made genome engineering a more accessible tool is CRISPR/Cas9 technology (Doudna and Charpentier, 2014). CRISPR/Cas9-induced gene Knock-Out (KO) is a highly efficient and relatively simple process that has been broadly applied not only in basic research but also in high-throughput drug screening (Blanck et al., 2020; Bock et al., 2022). Using CRISPR/Cas9 to introduce or Knock-In (KI) a DNA sequence is a more complex and laborious process; it is much less efficient than KO and requires more quality control steps and validation, especially for pluripotent stem cells (Ding et al., 2013; Yang et al., 2013; Zhu et al., 2015). A very useful KI application is the creation of fluorescently tagged fusion proteins. These reporter molecules allow for the tracking of expression levels and subcellular localization of the protein of interest. Tagged proteins with cell-type specific expression can serve as a marker to follow the differentiation and behavior of that particular cell population. There have been a number of recently detailed protocols describing this application (Koch et al., 2018; Sharma et al., 2018; Paix et al., 2019) but generating stem cells with integrated reporters or fusion proteins is still a challenge. Here, we describe an efficient method to deliver foreign DNA (plasmids containing Cas9/gRNA and homology arms sequences) into hiPSCs to insert fluorescent fusion tags. We show that our protocol delivers high rates of tag incorporation, allowing us not only to screen fewer individual clones but also to expand and characterize several homozygous KIs within the first round of transfection. We fused a super-fold green fluorescent protein (sfGFP) tag into the *PLP1* gene, an OLs marker, and characterized its performance using our 3D brain model. We showed the potential of this model as a screening tool for toxicants as well as a new tool to study OLs’ differentiation, migration, and myelination, which are very challenging processes to recapitulate *in vitro*.

## 2. Materials and methods

### 2.1. iPSC culture and quality control (QC)

Following Good Cell Culture Practice Guidance 2.0 document (Pamies et al., 2017b,2018b,^2020^, ^2022^), hiPSC culture quality control included periodic pluripotency testing (examination of colony morphology, flow cytometry to assess enrichment of pluripotency markers Oct4/Nanog/TRA1-60). Genetic stability was controlled every 10 passages using the qPCR-based hPSC Genetic Analysis kit (StemCell Technologies™). We used two different hiPS cell lines to demonstrate the reproducibility of the protocol: NIBSC8 (passage 16–20, female origin, kindly provided by National Institute for Biological Standards and Control, NIBSC, UK) and CS2PFYiCTR-nx.x (CS2, passage 27–30, male origin from Cedars-Sinai Medical Center Stem Cell repository). Both cell lines were authenticated by Short Tandem Repeat profiling and confirmed to be free from mycoplasma contamination by PCR. hiPSCs were cultured in mTeSR™ Plus medium (StemCell Technologies™) on vitronectin-coated plates at 37°C, 5% CO_2_ and 5% O_2_.

### 2.2. Plasmid design, generation, and isolation

A guide sequence targeting the stop codon of the *PLP1* locus was designed using DeskGen genome editing software (Desktop Genetics Ltd.). A guide sequence with minimal off-target and very high activity score was chosen and cloned into the *Bbs*I restriction site of the Cas9 plasmid (Cas9-P2A-Puro modified from Addgene #62988, Li et al., 2022). pSpCas9(BB)-2A-Puro (PX459) V2.0 was a gift from Feng Zhang (Addgene plasmid # 62988^1^). To clone the donor plasmid, a ∼2 kb PCR product was amplified from genomic DNA extracted from H9 ES cells and cloned into Zero Blunt TOPO cloning vector (Thermo Fisher Scientific). The sfGFP reporter DNA sequence was then introduced into the intermediate donor plasmid, precisely upstream of the *PLP1* stop codon, using Gibson assembly (New England Biolabs, Li et al., 2022). Plasmids were transformed in competent *Escherichia coli* (C4040-10, Invitrogen) and purified using Endotoxin-free Maxi-prep (Qiagen), following manufacturers’ protocols.

### 2.3. Transfection and transient puromycin selection

For reverse transfection, hiPSCs were grown in 6-well plates until 80% confluency. On the day of transfection, the following tubes were prepared: 25 μl of OptiMEM (Thermo Fisher Scientific), 176 ng of gRNA-containing plasmid, and 374 ng of *PLP1* template-containing plasmid were mixed in tube 1. Tube 2 contained 25 μl of OptiMEM and a plasmid carrying Cherry fluorescent protein as a control for transfection efficiency. Tube 3 contained 50 μl of OptiMEM and 3 μl of Lipofectamine Stem Transfection Reagent (Thermo Fisher Scientific). Cells were washed twice with 1X PBS and incubated with 1 ml of gentle cell dissociation reagent (GCDR, StemCell Technologies™) for 5 min at 37°C. During this time, the content of tube 3 was transferred into tubes 1 and 2 (25 μl each), pipetted up and down, and incubated for 10 min at RT. After 5 min incubation in GCDR, when gaps began to appear on the edges of the iPSC colonies, GCDR was carefully removed and 1 ml of mTeSR™ Plus medium was added and pipetted up and down several times with a P1000 pipette until clumps of about 3-5 cells were formed (observed under the microscope). Cells were transferred to a 15-ml tube, containing 3 ml of mTeSR™ Plus medium with 10 μM γ-27632 and counted. A total of 8 × 10^4^ cells/well were plated in 500 μl of mTeSR™ Plus with 10 μM γ-27632 in a Corning^®^ Matrigel^®^-coated 24-well plate. The content of tubes 1 and 2 was added dropwise to designated wells immediately after cell seeding. Three wells were seeded per cell line: (i) CRISPR/Cas9 KI constructs, (ii) Cherry plasmid transfection control, and (iii) non-transfected control. Forward transfection was performed following the same steps with the exception that cells were seeded the day before and the complexes were added when the cells were 60% confluent. After the addition of DNA complexes, cells were returned to the incubator for about 16 h. The next day, the medium was changed to mTESR™ Plus. Cells were allowed to grow and recover for an additional 24 h before transfectants were selected with 0.8 μg/ml puromycin. After 24 h of puromycin selection, surviving cells were washed twice with 1X PBS and cultured for 48 h to reach 50% confluency in mTESR™ Plus medium. Images to quantify transfection efficiency from forward and reverse transfection were taken in the Cherry-transfected well 48 h after transfection using the Revolve microscope (ECHO).

### 2.4. Single-cell colony formation and clonal expansion

Following the recovery period after transfection and puromycin selection, the single-cell suspension was prepared by incubating the cells with 300 μl of GCDR for 5 min at 37°C and pipetting up and down with a P1000 pipette 5–7 times. Single-cell suspension was transferred to a 1.5-ml tube containing 700 μl of mTeSR™ Plus supplemented with CloneR™ (StemCell Technologies™). Cells were counted and a 1:10 dilution was prepared using mTeSR™ Plus + CloneR™. The remaining cells were seeded in one well of a vitronectin-coated 24-well plate as transfection efficiency control. ∼330 cells from the cell suspension were added to 11 ml of medium + CloneR™ to prepare the final dilution of 30 cells/ml. This cell suspension was thoroughly mixed and seeded into two 96-well plates previously coated with vitronectin (50 uL/well) aiming to get 1 cell/well. After 48 h, 100 μl of mTeSR™ Plus medium + CloneR™ were added to both plates, all wells were screened and only those containing a single colony of 4–8 cells were selected. 96 h after seeding, the medium was changed to mTeSR™ Plus. Colonies were allowed to grow for 7–10 days and then split into two 48-well plates as follows. A total of 100 and 150 μl of mTeSR™ plus with 10 μM y-27632 were added to vitronectin-coated 48-well plates 1 and 2, respectively. A total of 30 μl GCDR were added to each well of a 96-well plate and incubated for 5 min at 37°C. GCDR was removed and 200 μl of mTeSR™ Plus medium with 10 μM Y-27632 was added to each well and cells were pipetted up and down three times. Aliquots of either 150 or 80 μl of the cell suspension were transferred to 48-well plates 1 and 2, respectively. Plate 1 was then used for DNA isolation and KI screening and validation, while plate 2 was used to generate the cell bank of the candidate clones.

### 2.5. Knock-in screening and validation

*PCR and genomic sequencing:* DNA isolation of transfectant pool or from single cell-derived colonies was performed using Quick-DNA Miniprep Kit (Zymo Research). DNA quantity and quality were determined using NanoDrop™ 2000c Spectrophotometer (Thermo Fisher Scientific). A total of 50 ng of DNA were amplified in a 20 μl PCR reaction using Phusion Flash High-Fidelity PCR Master Mix (Thermo Fisher Scientific) to assess transfection efficiency (Primer sequences are listed in Supplementary Table 1). Primer melting temperatures were calculated using the Thermo Fisher Scientific Tm calculator and extension time was assigned depending on product size (15s/1Kb). PCR products were visualized on 1.5% agarose gel containing SYBR Safe DNA Gel Stain (Thermo Fisher Scientific). Up to 5 transfectants containing homozygous insertion were then sequenced by Sanger sequencing to detect possible mutations in the insertion area and homology arms used (Primer sequences are listed in Supplementary Table 1). Briefly, 2 μl of the template were mixed with 2 μl of 1 μM primers, then 2 μl of this mixture was added to the standard sequencing master mix (the standard master mix is a 1/16 dilution of the master mix in the Applied Biosystems instrument protocols). PCR amplification step was performed using the Applied Biosystems 3730 instrument with the 2019 upgrade. The sequencing reactions were performed in Verti Thermocycler (Thermo Fisher Scientific) and an Optima DTR 96-Well plate (Edge Biosystems) was used to clean up the reaction after thermocycling.

*Off-target effects analysis:* CCTop online tool^2^ was used to identify the top 10 potential off-target sites of the gRNA/Cas9 construct. Sanger sequencing was performed as described for insert sequencing (Primer sequences are listed in Supplementary Table 2).

*Chromosome aberration analysis by RT-PCR and short tandem repeats (STR):* DNA was extracted using PureLink Genomic DNA Mini Kit (Thermo Fisher Scientific) and karyotypic abnormalities frequently reported in human ES and iPS cells were assessed with the hPSC Genetic Analysis Kit (StemCell Technologies™) following manufacturer instructions. Quantitative Real-Time PCR (RT-qPCR) was performed using the CFX96 Touch Real-Time PCR Detection System (Bio-Rad). For authentication by STR analysis, a Promega PowerPlex 18D Kit was used to amplify 17 short tandem repeat (STR) loci plus gender-determining marker Amelogenin. The PCR products were electrophoresed on an ABI Prism^®^ 3730xl Genetic Analyzer using the internal lane standard WEN-ILS-500 (Promega). Positive and negative controls were 2800M Control DNA (Promega) and water, respectively. Data were analyzed using GeneMapper^®^ version 5.0 software (Applied Biosystems).

### 2.6. Neural progenitor cell (NPC) and bMPS generation

NPC generation and expansion from hiPSC were performed using the PSC Neural Induction medium following the manufacturer’s protocol (Thermo Fisher Scientific). Briefly, 2 × 10^5^ iPSC were seeded as small colonies per well in a matrigel-coated 6-well plate in mTeSR™ Plus medium supplemented with 10 μM y-27632. The next day medium was changed to neural induction medium (Neurobasal medium and 1X neural induction supplement). After 7 days of neural induction, NPC colonies were dissociated and expanded in the Neural Expansion medium (50% Neurobasal, 50% Advanced DMEM/F12 media, 1X Neural induction supplement, and 5 μM y-27632) for at least three passages before freezing the NPC stock. Y-27632 was removed the next day after passaging. After passage four, NPCs were cultured without y-27632. Cultures were maintained at 37°C, with 5% CO_2_ and 5% O_2_, with every second-day medium change. Passages 6–12 were used to generate bMPS. bMPS were generated as described before (Pamies et al., 2017a). Briefly, 90–100% confluent NPCs were washed with PBS and treated with GCDR for 5 min at RT. GCDR was removed, fresh Neural Expansion medium was added, and NPCs were detached mechanically by scratching and counted. A total of 2 × 10^6^ cells per well were plated in uncoated 6-well plates. After 2 days, Neural Expansion medium was changed to differentiation medium (B-27™ Plus Neuronal Culture System or B-27™ Electrophysiology kit, 1% Glutamax (Gibco, Thermo Fisher Scientific), 0.01 μg/ml human recombinant GDNF (GeminiBio™), 0.01 μg/ml human recombinant BDNF (GeminiBio™), 1% Pen/Strep (Gibco, Thermo Fisher Scientific). Cultures were kept at 37°C, 5% CO_2_, and 20% O_2_ under constant gyratory shaking (88 rpm, 19 mm orbit) for up to 10 weeks. About 75% of the medium was changed three times a week. For cuprizone experiments, 8-week-old bMPS were exposed to 25 μM cuprizone (CPZ, Sigma Aldrich) for 7 days. DMSO was used as vehicle control.

### 2.7. RNA extraction and RT-qPCR

Total RNA was extracted using the Quick-RNA Microprep Kit (Zymo Research). RNA quantity and purity were determined using NanoDrop™ 2000c Spectrophotometer (Thermo Fisher Scientific). A total of 500 ng of RNA were reverse-transcribed using M-MLV Reverse Transcriptase and Random Hexamer primers (Promega) according to the manufacturer’s instructions. The final reaction was diluted 5x with molecular grade RNAse/DNAse-free water. Gene expression was evaluated using TaqMan gene expression assays (Supplementary Table 3, Thermo Fisher Scientific). RT-qPCR was performed using the CFX96 Touch Real-Time PCR Detection System (Bio-Rad). The 2^–ΔΔCt^ method (Livak and Schmittgen, 2001) was used to calculate the relative gene expression changes over time of neural differentiation or due to treatment with cuprizone. Gene expression was normalized to the expression level at the NPC stage and 18S rRNA expression was used as the housekeeping gene.

### 2.8. Immunofluorescence

bMPS were collected in 1.5-ml tubes, washed with 1X PBS, and fixed with 4% paraformaldehyde (PFA, Sigma Aldrich) for 45 min at 4°C, washed twice with 1X PBS and permeabilized with 1 ml permeabilization solution (1X PBS, 0.1% Triton X) for 30 min at 4°C. Cultures transferred to a 24-well plate and blocked with 300 μl of 100% BlockAid (Thermo Fisher Scientific) on a shaker for 1 h at 4°C. Primary antibodies (Supplementary Table 4) were diluted in antibody staining solution (1X PBS, 10% BlockAid, 0.1% Triton-X, 0.5% BSA); 200 μl added to each well and incubated overnight at 4°C on a shaker. Then, cultures were washed three times with 1 ml of organoid washing buffer (OWB: 1X PBS, 0.1% Triton-X, 0.5% BSA) for 1 h each washing step at 4°C on a shaker. Secondary antibodies (Goat-anti mouse or rabbit AlexaFluor^®^ 568 and 647, Thermo Fisher Scientific) and nuclei staining (Hoechst 33342, Invitrogen) were prepared in antibody staining solution and spun down at 10,000 × *g*, for 10 min at 4°C). A total of 200 μl of secondary antibodies were added per well and bMPS were incubated overnight at 4°C on a shaker. Washing steps were repeated three times with OWB for 1 h each. After the third wash, bMPS were mounted on a glass slide using ImmuMount medium (Thermo Fisher Scientific), a single layer of double-sided sticky tape (Scotch 3M), and a PAP pen (ImmEdge™ Pen, Vector Laboratories). Slides were imaged using ZEISS LSM800 confocal microscope. Image processing and quantification were performed using Zen 3.3 blue edition (ZEISS), Imaris software version 9.8.2 (Bitplane AG), and ImageJ version 2.0.0-rc-69/1.52p.

### 2.9. Transmission electron microscopy (TEM)

Samples were fixed in 2.5% glutaraldehyde, 3mM MgCl_2_, in 0.1 M sodium cacodylate buffer, pH 7.2 overnight at room temperature. After buffer rinse, samples were postfixed for 2 h in 1.5% potassium ferrocyanide, reduced 2% osmium tetroxide in 0.1 M sodium cacodylate on ice in the dark. Following a dH_2_O rinse, samples were incubated in 1% uranyl acetate (UA) for 2 h at 4°C, followed by graded dehydration of ethanol and propylene oxide. Samples were embedded in EPON mixture and polymerized at 60°C overnight. Thin sections, 60–90 nm, were cut with a diamond knife on a Leica Ultracut UCT ultramicrotome and picked up with 2 × 1 mm formvar coated copper slot grid. Grids were observed on a Hitachi 7600 TEM at 80 kV and images captured with an AMT CCD XR80 (8-megapixel camera – side mount AMT XR80 – high-resolution high-speed camera).

### 2.10. bMPS dissociation and flow cytometry

bMPS were dissociated using the Papain Dissociation kit (Worthington Biochemical). Briefly, bMPS were washed twice with Hibernate E medium (Thermo Fisher Scientific), the medium was aspirated, and cultures were resuspended in 0.7 ml of papain/DNAse solution containing 20 U of papain and 100 U of DNAse per ml in 1 mM L-cysteine with 0.5 mM EDTA. Organoids were incubated for up to 3 h at 37°C on a shaker. Every 30 min, the cultures were pipetted up and down with a P1000 pipette 3–4 times to accelerate dissociation. After full dissociation, 1 ml of Hibernate E medium was added, and the cell suspension was collected into flow cytometry tubes (Corning™Falcon™) and spun down at 300 × *g* for 4 min at RT. Then, the pellet was resuspended in 0.7 ml albumin-ovomucoid solution and incubated for 10 min at RT. The tubes were centrifuged at 300 × *g* for 4 min at RT. The pellet was resuspended in 1 ml of 1X PBS and 1 μl of Fixable Viability Stain 780 (BD Horizon) and incubated for 5 min at 37°C protected from light. After incubation, 2 ml of Stain Buffer (BD Biosciences) were added and cells were centrifuged at 300 × *g* for 4 min at RT.

Pellet was then resuspended in 500 μl of 2% PFA (Sigma Aldrich) and cells were fixed for 20 min at 4°C. A total of 500 μl of permeabilization solution [1X PBS, 1% Triton™ X-100 (Sigma-Aldrich)] were then added for 10 min at RT. A total of 2 ml of washing solution 1 (WS1: 1X PBS, 1% (BSA, Sigma), 0.1% Triton X) was then added to the cells and centrifuged at 300 × *g* for 4 min at 4°C. The cells pellet was resuspended in 300 μl of blocking solution (1X PBS, 1% BSA, 10% normal goat serum, 0.1% Triton™ X-100) and incubated for 1 h at 4°C. A total of 1 ml of WS1 was added and samples were centrifuged at 300 × *g* for 4 min at 4°C. The cells were then resuspended in 100 μl of blocking solution and conjugated antibodies (Supplementary Table 5) were added directly to each tube and incubated for 1 h at 4°C in the dark. Next, 2 ml of WS1 were added to each sample and cells were centrifuged at 300 × *g* for 4 min at 4°C. The wash step was repeated once. For the third wash, 2 ml of washing solution 2 (WS2: 1X PBS, 1% BSA) was used. The cells were then resuspended in 300 μl of WS2 and analyzed on the flow cytometer (SONY SH800). For compensation and gating, single-color controls were included for each experiment. Unstained cells were used as a control for endogenous fluorescence and viability, and compensation beads were used for antibody stainings [anti-mouse Ig, k/negative control Compensation Particles set, BD Biosciences, and UltraComp eBeads™ Compensation Beads (Thermo Fisher Scientific)]. Compensation was performed before running the samples and later when processing the results. Flow cytometry data was processed and visualized using FlowJo 10.8.1 (Becton Dickinson & Company) and the gating hierarchy for each flow cytometry experiment is presented in Supplementary Figure 7.

### 2.11. Statistical analysis

**FIGURE 1:**
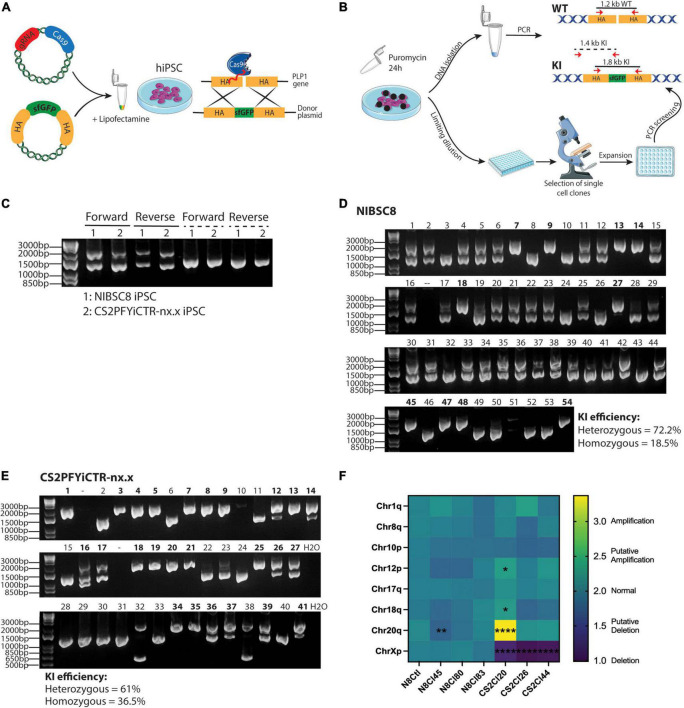
CRISPR/Cas9 gene-editing of PLP1-sfGFP fusion protein in hiPSCs. **(A)** Schematic representation of the method used to incorporate the fluorescent tag into hiPSCs (HA, Homology arms). **(B)** Scheme showing puromycin selection of cells containing the gRNA/Cas9 plasmid followed by cell seeding, expansion, and preliminary screening of the clones. Annealing sites of primers used for screening are shown with red arrows and the sizes of the amplicons are denoted. WT, wild type; KI, Knock In. Drawings were created using SMART art; https://smart.servier.com/. **(C)** Agarose gel showing KI efficiency of forward and reverse transfections in the transfectant pools of the two hiPSC lines. Solid lines show the amplicons when primers were located inside the HA [as shown in panel **(B)**] and dashed lines show amplification regions when the forward primer targeted *PLP1* sequence outside the HA region and the reverse primer bound to sfGFP sequence [as pictured in panel **(B)**]. Agarose gel showing 54 and 41 single-cell clones for NIBSC8 **(D)** and CS2 **(E)** after reverse transfection. Double bands show heterozygous insertion; an individual band at 1,800 bp suggests homozygous insertion (clone IDs marked bold); and an individual band at 1,200 bp suggests wild type without insertion. **(F)** Heatmap showing the expression of the 8 most common karyotypic abnormalities reported in hiPSCs. CS2PFYiCTR-nx.x clones show deletion of chromosome X due to gender difference (male) when compared to the control iPSCs (NIBSC8 wildtype, female). Statistical significance was calculated using two-way ANOVA with Sidak’s multiple comparison test.*p < 0.05, **p < 0.01, ****p < 0.0001.

All the graphs and statistical analysis were done in GraphPad Prism, version 9. Data sets were tested for normal distribution using the Shapiro-Wilk test. Data sets showing normal distribution were analyzed using unpaired, two-tailed *t*-test (Figures 6A, B, D, E and Supplementary Figures 5C–E) and unpaired, one-tailed *t*-test (Figure 6G). Grouped analysis and multiple comparisons were analyzed using two-way ANOVA with Šidák’s multiple comparison test (Figures 1F, 3C, 6F). “*n*” represents the number of independent experiments (biological replicates). *P* < 0.05 was considered significant.

**FIGURE 2:**
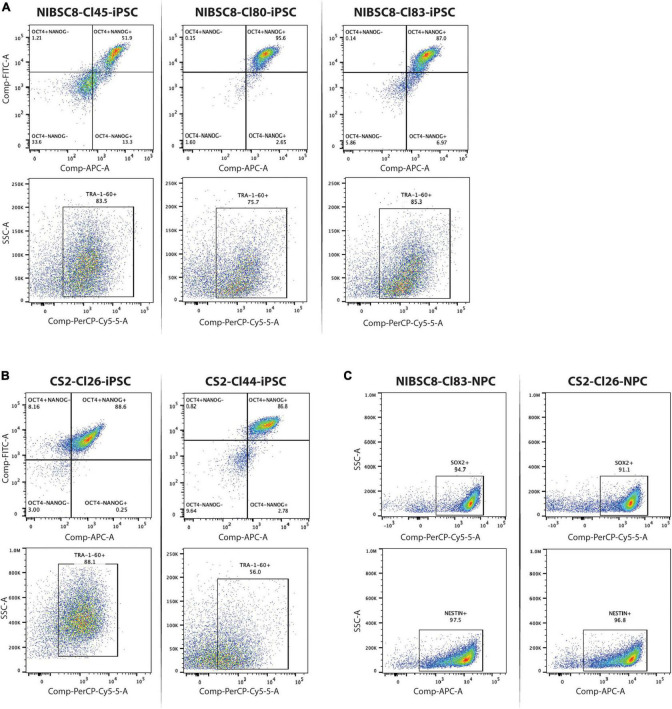
Expression of stem cell and neuroprogenitor cell markers in selected clones containing sfGFP tag. Flow cytometry charts showing the expression of three hiPSC markers (OCT4, NANOG, TRA1-60) in the selected clones for NIBSC8 **(A)** and CS2 **(B)** cell lines. **(C)** Flow cytometry charts showing expression of neuroprogenitor cell markers (SOX2 and NESTIN) following neural induction of NIBSC8-Cl83 and CS2-Cl26 hiPSCs. Gates for both cell lines and clones were set up based on corresponding single color/unstained controls separately for each line/clone (Supplementary Figures 7A, B). SSC-side scatter.

**FIGURE 3:**
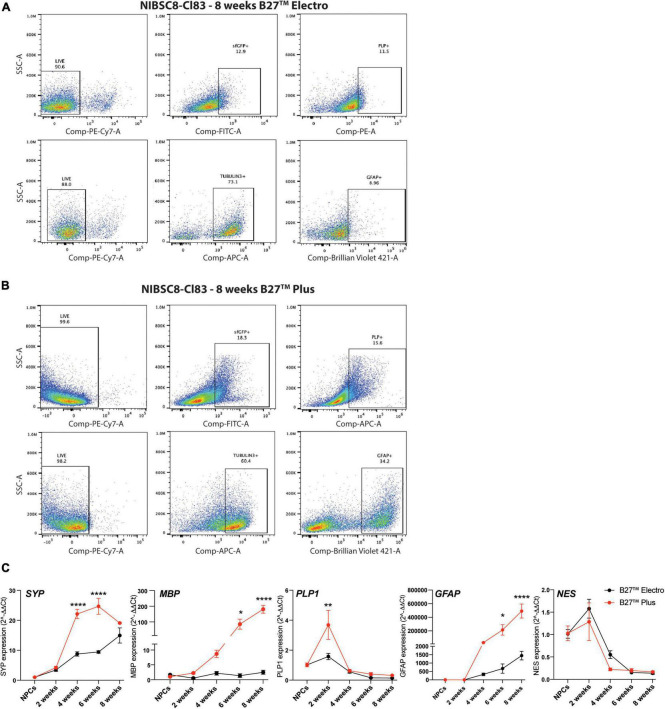
Optimization of neural differentiation. Representative flow cytometry charts showing sfGFP+, PLP+ and GFAP+ cells in 8-week-old NIBSC8-Cl83 bMPS cultured in B27™ Electro **(A)** and B27™ Plus media **(B)**. Gates for both conditions were set up based on corresponding single color/unstained controls separately for each condition (Supplementary Figures 7E, F). **(C)** Gene expression of neural markers in NIBSC8-Cl83 bMPS generated using B27™ Electro vs. B27™ Plus media. Statistical significance was calculated using two-way ANOVA with Šidák’s multiple comparison test. Data are represented as mean ± SEM from two independent experiments with 2–3 technical replicates. *p < 0.05, **p < 0.005, ****p < 0.0001. SSC, side scatter; SYP, synaptophysin; MBP, myelin basic protein; PLP1, proteolipid protein 1; GFAP, Glial Fibrillary Acidic Protein; NES, Nestin.

**FIGURE 4:**
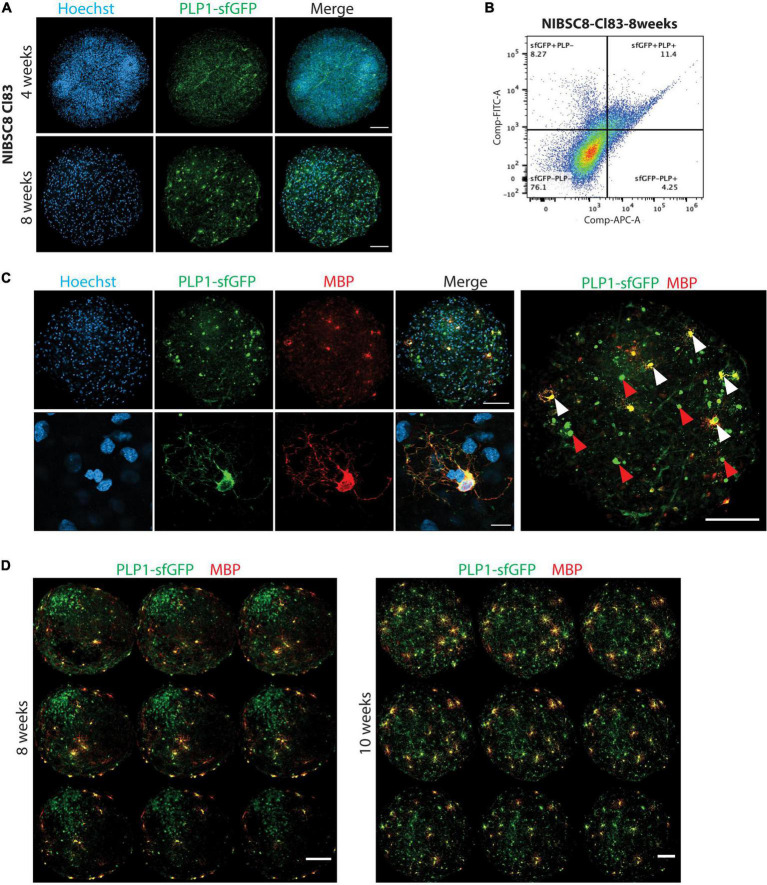
sfGFP specific expression and intracellular co-localization with OLs in bMPS derived from NIBSC8-Cl83 hiPSCs. **(A)** Representative confocal images of 4- and 8-week-old bMPS showing the sfGFP+ cells (green) in the organoids. Different morphology was observed at 4 vs. 8 weeks. Nuclei are stained with Hoechst (blue, scale bar: 100 μm). 5–8 bMPS were screened per week per experiment in three independent experiments. **(B)** A representative flow cytometry chart from three independent experiments shows co-localization of PLP1 (antibody labeling) and sfGFP (endogenous) in 8-week-old bMPS. Gates for both conditions were set up based on corresponding single color/unstained controls separately for each condition (Supplementary Figure 7F). **(C)** Representative confocal images of 8-week-old bMPS showing PLP1-sfGFP (green) and MBP (red) expression in OLs. White arrowheads show mature OLs (sfGFP+/MBP+) and red arrowheads show immature OLs (GFP+/MBP-; scale bar: upper panel 100 μm; lower panel 10 μm). Nuclei are stained with Hoechst (blue). 5–8 bMPS were screened per experiment in three independent experiments **(D)** Representative montage of z-stack imaging in 8- and 10-week-old bMPS showing immature OLs (GFP+MBP-) and OLs (sfGFP+ MBP+) migration and maturation (scale bar: 100 μm). 5–8 bMPS were screened per week per experiment in three independent experiments.

**FIGURE 5:**
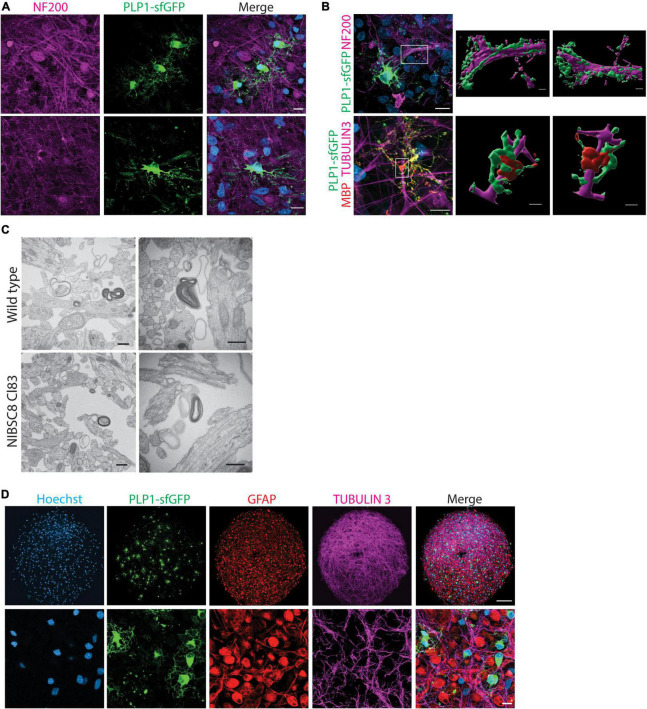
Functional validation of PLP1-sfGFP fluorescent tag and other cell types present in the bMPS. **(A)** Representative confocal images of 8-week-bMPS derived from NIBSC8-Cl83 hiPSCs showing sfGFP+ cells (green) with distinct OLs morphology and neuronal axons stained with antibody against neurofilament protein (NF200, magenta). The lower panel displays maximal projection intensity images showing PLP1 located alongside axons. **(B)** Front- and rear-view of a 3D reconstruction from confocal z-stacks demonstrating PLP1-sfGFP interaction with axons at 8 weeks (upper panel) and MPB and PLP1-sfGFP wrapping around the axons at 10 weeks (lower panel). The color segregation in the image is an artifact of the imaging processing due to single channel 3D rendering; scale bars: 10 μm, 3D images 2 μm. **(C)** Representative TEM images of myelinated axons in wildtype and NIBSC8-Cl83 bMPS (scale bars: 500 nm). **(D)** Representative confocal images in lower and higher magnifications of 8-week-old NIBSC8-Cl83 bMPS showing neurons (TUBULIN 3, magenta), astrocytes (GFAP, red), and oligodendrocytes (sfGFP, green) markers. The lower panel shows a maximal projection intensity image (scale bar: upper panel 100 μm; lower panel 10 μm). A total of 5–8 bMPS were screened per experiment in three independent experiments.

**FIGURE 6:**
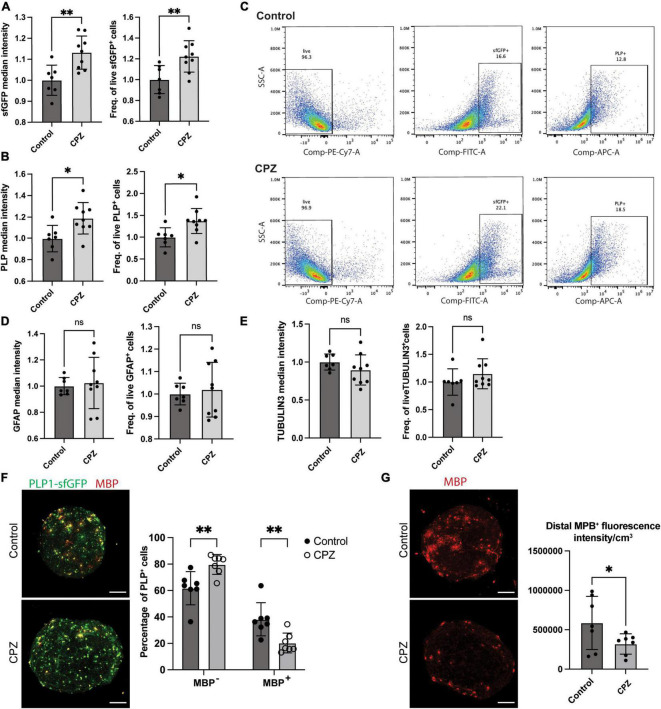
Modulation of the PLP-sfGFP tag by CPZ in NIBSC8-Cl83 bMPS. **(A,B)** Increased sfGFP and PLP1 median intensity and the number of positive cells assessed by flow cytometry in 9-week-old bMPS after 7 day-exposure to 25 μM CPZ vs. vehicle control (DMSO). Statistical significance was calculated using unpaired, two-tailed *t*-test. Data represent the mean ± SD, from 3 independent experiments with 2–3 technical replicates per experiment. **(C)** Representative flow cytometry plots showing the percentage of live, sfGFP+ and PLP1+ cells in Control and CPZ-treated bMPS. Gates were set up based on corresponding single color/unstained controls (Supplementary Figure 7G). **(D,E)** Unchanged TUBULIN 3 and GFAP median intensity and number of positive cells assessed by flow cytometry in 9-week-bMPS after 7 day-exposure to CPZ vs. vehicle control. Statistical significance was calculated using unpaired, two-tailed *t*-test. Data represent the mean ± SD, from 3 independent experiments with 2–3 technical replicates per experiment. Gates were set up based on corresponding single color/unstained controls (Supplementary Figure 7G). **(F)** Representative z-stacks of PLP1-sfGFP 9-week old bMPS stained with MBP antibody after treatment with CPZ or vehicle control. The chart on the right shows the quantification of immature OLs (GFP+ MBP-) and mature OLs (GFP+ MBP+) using Imaris software (7 bMPS/condition, scale bar: 100 μm). Statistical significance was calculated using two-way ANOVA with Sidak’s multiple comparison test. **(G)** Representative z-stacks of PLP1-sfGFP 9-week old bMPS stained with MBP antibody after treatment with CPZ or vehicle control. The chart on the right shows the quantification of MBP overall intensity subtracting cell bodies using Image J (7 bMPS/condition, scale bar: 100 μm). Statistical significance was calculated using unpaired, one-tailed *t*-test. *p < 0.05, ^**^p < 0.01.

## 3. Results

### 3.1. Reverse transfection and transient antibiotic selection improve tag incorporation efficiency

To follow oligodendrocyte maturation, we introduced a sfGFP tag just before the stop codon of *PLP1* in two hiPSC lines. We used female (NIBSC8) and male (CS2PFYiCTR-nx.x) iPSC lines. The fusion was designed to include a short serine/glycine linker peptide between the coding region for *PLP1* and *sfGFP* (Supplementary Figure 1A), as it is suggested to allow proper folding of the fluorescent protein (Chen et al., 2013; Sluch et al., 2018; Li et al., 2022). This strategy of insertion of the tag as a fusion also allowed the coupling of the GFP tag to PLP1 protein, so that the PLP1 cellular localization can be followed without any alterations in PLP1 functions as described previously for this specific tag (Li et al., 2022). We used two different methods to introduce the targeting gRNA and the donor template for homology-directed repair to incorporate the fusion: reverse and forward transfections (Figure 1A). We did not observe a difference in transfection efficiency between the two methods (Supplementary Figure 1B). To increase the efficiency of selecting clones with the desired integration, we used a Cas9/gRNA plasmid that contains a puromycin resistance sequence, since it was previously shown to increase the KI efficiency of reporter genes (Sluch et al., 2018). Following puromycin selection, transfection efficiency was estimated by PCR in DNA extracted from the pool of transfected cells using two sets of primers. The first set was complementary to the homology arms (HA) used to insert the tag (Figures 1B, C, solid lines). The lower band (1,200 bp) shows wild type (WT) while the upper band (1,800 bp) successful KI. The second set of primers, binding a genomic region outside the HA and *sfGFP* sequence, was designed to detect only the incorporation of the tag into the genome (dashed lines in Figures 1B, C). We observed overall high transfection efficiency in both cell lines (Figure 1C). The second primer set was used to confirm successful incorporation into the genome since the first set could potentially amplify residual template donor plasmid. Following limiting dilution seeding, about 50 single cell clones were expanded, and PCR was performed to screen for heterozygous and homozygous KI. Forward transfection yielded 46% heterozygous incorporation and only 3.3% homozygous incorporation in the screened colonies (data not shown). With reverse transfection, however, both cell lines showed a high percentage of heterozygous tag incorporation (72.2 and 61% for NIBSC8 and CS2, respectively), but more striking was the percentage of homozygous incorporation of the tag (19-37%, Figures 1D, E). These findings strongly suggest an improvement in tag incorporation when using reverse transfection in difficult-to-transfect hiPSCs.

### 3.2. Quality control of transfectants: Detection of off-target effects and chromosome aberrations in the PLP1-sfGFP hiPSC clones

To verify the KI, we performed Sanger sequencing of the *sfGFP* insert and homology arms in 4-5 clones for each cell line. The incorporation occurred without any mutations, as shown in representative alignment (Supplementary Figure 1C). Next, we used CCTop online tool to identify the top 10 potential off-target sites for the gRNA used (Supplementary Figure 1D). These 10 regions were sequenced in the same clones. Out of the 10 regions, only one gene (*IFT52*) showed off-target mutation with a 25-40% penetrance (data not shown and those clones were discontinued). After the off-target screening, 3 clones per cell line were analyzed for chromosomal aberrations in the most common mutation sites identified for hiPSCs. This assay identified one putative deletion in NIBSC8 line clone 45 as well as one amplification and two putative amplifications in CS2 line clone 20 (Figure 1F). These clones were discontinued. Simultaneously, characterization of the pluripotency markers OCT4, NANOG, and TRA-1-60 by flow cytometry showed satisfactory enrichment (>85%) in NIBSC8 line clone 83 and CS2 line clone 26 (Figures 2A, B). STR profiling for these clones showed a 100% identity match with the corresponding wild type and their cell line of origin (MRC-9 for NIBSC8 and NEUMT932PF for CS2, Supplementary Figures 1E–H). Based on the hiPSC QC, clones 83 and 26 were selected for the next steps of NIBSC8 and CS2 cell line validation, respectively.

### 3.3. Optimization of neural differentiation

We differentiated the two selected PLP1-sfGFP hiPSC clones to NPCs and characterized the differentiation efficiency by flow cytometry using neural stem cell markers, SOX2 and NESTIN (Lendahl et al., 1990; Ellis et al., 2004; Latremoliere et al., 2018). Over 90% of the cells were NESTIN- and SOX2-positive in both cell lines, confirming the purity of the NPC cultures (Figure 2C). This was followed by differentiation to bMPS, as previously described by our group (Pamies et al., 2017a). We compared the cellular profile, of organoids differentiated using the B-27™ Plus Neuronal Culture System vs. the B-27™ Electrophysiology kit, as the Plus medium was suggested by the manufacturer to provide an optimized formulation to support neural survival and network formation. When compared to Electro medium, at 8 weeks of differentiation Plus medium enriched populations of OLs (sfGFP^+^, PLP1^+^) and astrocytes (Glial fibrillary acidic protein, GFAP^+^) from ∼13 to ∼18% and ∼9 to ∼34%, respectively (Figures 3A, B). Supporting these results, gene expression analysis showed an increased expression of *MBP* (OL marker) and *GFAP* in 8-week-old bMPS cultured with Plus vs. Electro media (Figure 3C). Neuronal and synaptic marker, Synaptophysin (*SYP*) was expressed significantly higher at 4 and 6 weeks in organoids cultured in Plus medium, however, the organoids in Electro medium reached the same level of *SYP* expression by week 8. An NPC marker, *NES*, showed a steeper decrease in Plus medium than in Electro (Figure 3C). These data suggest that the Plus medium induces neurogenesis faster and supports more glia lineage differentiation. Thus, we show experiments performed in Plus medium in the main figures and report respective key experiments done in Electro as Supplementary material.

### 3.4. Functional validation of the fusion cell lines in bMPS

We followed the expression of sfGFP upon differentiation of NPCs to bMPS over time in both cell lines. Strikingly, both selected clones, NIBSC8-Cl83 and CS2-Cl26, showed green fluorescence when evaluated at 4 and 8 weeks of differentiation (Figure 4A and Supplementary Figures 2A, B). We observed characteristic OL morphology at 8 weeks together with long processes. In addition, gene expression of *PLP1* and *sfGFP* tag showed a significant positive correlation throughout the development of the bMPS (NIBSC8-Cl83 R = 0.9998; CS2-Cl26 R = 0.8806; Supplementary Figures 2C, D). To further assess the tag specificity, we performed flow cytometry to measure colocalization between endogenous PLP1-sfGFP and antibody-detected PLP1. In 8-week-old bMPS, differentiated in Plus medium, ∼16% of live cells were stained with PLP1-antibody and ∼19% of live cells were GFP^+^, with 73% overlap between endogenous GFP^+^ and PLP1^+^ cells (Figure 4B). bMPS differentiated with Electro medium showed 70% overlap between PLP1^+^ and GFP^+^ cells in NIBSC8-Cl83 and 55% for CS2-Cl26 (Supplementary Figures 2E, F).

Next, immunofluorescent staining of the bMPS was performed to further characterize the oligodendrocyte population and confirm tag specificity in our model. In addition to challenges associated with the whole-mount organoid immunostaining and imaging (Dekkers et al., 2019; Albanese et al., 2020), the limited permeability of the PLP1 antibody to efficiently detect the protein in a 3D context was another reason to create the fluorescent tag in the *PLP1* gene. Supplementary Figure 3A shows representative images of PLP1 antibody labeling in the 3D bMPS, which co-localized with endogenous GFP signal, but showed a high level of background and low signal. To further ensure that the PLP1-sfGFP tag is specific for OLs, we included a co-staining with MBP, the major myelin protein from the nervous system, and a marker of mature OLs. At 8 weeks, MBP-positive cells showed OL morphology with multiple branches or extended processes and a strong colocalization with cells endogenously expressing the sfGFP tag (Figure 4C and Supplementary Figures 3B, C). In addition, bMPS differentiated in Plus medium showed the distinction of immature OLs (GFP^+^, MBP^–^ cells with 0 to few branches, red arrowheads in Figure 4C) from mature OLs (GFP^+^, MBP^+^ cells with extensive branching; white arrowheads in Figure 4C).

Furthermore, qualitative assessment of 3D-imaging in NIBSC8-Cl83 bMPS using the z-stack application and MBP pseudo-staining revealed spatial differences in the localization of immature vs. mature OLs within the bMPS. Eight-week-old bMPS showed the presence of a core of immature OLs and a few mature OLs located distant from the core and mostly in the periphery of the organoid, strongly suggesting active migration before OL maturation (Figure 4D). At 10 weeks, the immature OL core disappeared, and these cells migrated out and were located near mature MBP^+^ OLs. The number of MBP^+^ cells also increased when compared to 8-week-old bMPS (Figure 4D). The immature OL agglomeration was also present at earlier stages of differentiation and in bMPS cultured with Electro medium (Supplementary Figure 2A).

Detection of neurofilaments (NF200 antibody) was used to label axons and elucidate their interactions with OLs in our model. We observed a PLP1-sfGFP signal along neural processes (Figure 5A and Supplementary Figures 4A, B) and 3D-reconstruction of NIBSC8-Cl83 bMPS at 8 and 10 weeks showed PLP/MBP wrapping around the neural processes (Figure 5B), suggesting the formation of myelin sheath in the bMPS. To confirm this, we performed transmission electron microscopy (TEM) in 10-week-old NIBSC8-Cl83 bMPS and identified myelin sheets around the axons (Figure 5C).

Finally, analysis of gene and protein expression levels of neuron and glia markers confirmed proper neural development of the clones. Gene expression showed an expected increase of Synaptophysin (*SYP*, synapsis marker), *MBP* (OL marker), and *GFAP* (astrocytes marker) and a decrease of *NES* (neural progenitor marker) during brain organoid’s development in both cell lines (Figure 3C and Supplementary Figure 4C). Evaluation of protein expression was performed by immunofluorescence and flow cytometry. Both cell lines showed the presence of neuronal and astrocyte markers as expected (Figures 3B, 5D and Supplementary Figures 4D–F, 5A). Taken together, these results suggest the normal functionality of the PLP1-sfGFP fusion protein as well as the absence of abnormalities or toxicities due to the fluorescent tag. This confirms the relevance and functionality of PLP1-sfGFP bMPS to be used to address oligodendrogenesis and its perturbation in a physiologically relevant environment.

### 3.5. Specific modulation of the PLP1-sfGFP tag by cuprizone (CPZ) treatment

Lastly, to demonstrate the versatility and applicability of the model to screen the effect of drugs and chemicals on OLs, we treated 8-week-old bMPS with 25 μM cuprizone (CPZ) for 1 week. CPZ is a copper chelator agent frequently used as a demyelination agent in murine models (Torkildsen et al., 2008; Skripuletz et al., 2013; Baxi et al., 2017). Surprisingly at first, flow cytometry analysis in CPZ-treated bMPS showed a significant increase in median intensity and number of positive cells for endogenous PLP1-sfGFP and antibody-labeled PLP1 (Figures 6A–C). There were no significant changes in the number of neurons or astrocytes after exposure to CPZ (Figures 6D, E and Supplementary Figure 5B). We also observed no CPZ-induced changes in gene expression of OL markers, *PLP1* and *MBP* (Supplementary Figure 5C), but *CSPG4*, one of the two OPC markers evaluated, was significantly increased (Supplementary Figure 5D). To follow up on this we quantified PDGFRα protein expression by immunofluorescence and in aggrement with qPCR data, did not observe changes between treatments (Supplementary Figure 5E). However, quantification of the number of GFP^+^/MPB^–^ cells (immature OLs) showed a significant increase after CPZ treatment with a concomitant decrease in the number of GFP^+^/MPB^+^ cells (mature OLs, Figure 6F). Furthermore, quantification of MBP expression only in the processes and not the cell bodies of mature OLs showed a decrease after CPZ treatment (Figure 6G and Supplementary Figure 6). This suggests a compensatory increase of immature OL generation in response to demyelination and loss of mature OLs observed after CPZ treatment in the selected treatment scheme.

## 4. Discussion

hiPSCs are a well-established source of versatile models, including recently emerged organoids. Although broadly used, there are several challenges associated with both hiPSC and 3D cultures (Zaaijer et al., 2021; Pistollato et al., 2022). Genetic manipulations of hiPSCs, especially the generation of reporter and fusion KI lines, is a technically difficult process, with much lower transfection efficiencies than those in immortalized cell lines, while robust and reproducible organoid differentiation remains a challenge as well. In addition, many endpoint measurements (particularly those based on imaging), which are well established for monolayer cultures, need significant optimization to be applied in 3D models. These two issues have been addressed in this study: genetic manipulation of hiPSC as well as imaging and lineage tracking in bMPS. Here, we described a very efficient method to deliver foreign DNA (plasmids containing Cas9/gRNA and homology arms sequences) into hiPSC to generate a fusion protein of interest. We took advantage of reverse transfection to increase the cellular surface area for the entrance of lipid vesicles and combine it with transient antibiotic resistance selection as described before (Sluch et al., 2018). We showed high rates of tag incorporation following this protocol (heterozygous up to 72% and homozygous up to 37%), which allowed us to expand and characterize several homozygous knock-in within the first round of transfection. Gene editing in hiPSC is known to be associated with an increased risk of genetic aberrations that can impact cellular homeostasis and differentiation potential (Mills et al., 2013; Zaaijer et al., 2021). Therefore, extensive QC is necessary to ensure a stable and reliable reporter line. Clone selection was performed based on karyotype aberration screening, sequencing of the inserted tag, gRNA/Cas9 off-target effects analysis, and validation of the cell line pluripotency and neural differentiation. We found two clones with possible karyotype aberrations, which is expected due to the high number of cell divisions after the limiting dilution seeding. Regarding off-target effects, we had one hit with an important penetrance (IFT52, 25-40%). These clones were discontinued. However, due to the high efficiency of tag incorporation, we had enough clones passing QC to continue with the functional validation. There are newer approaches to help reduce the number of off-target effects when using CRISPR/Cas9, e.g., the use of Ribonucleoproteins (RNPs) or improved Cas variants (Naeem et al., 2020), but those methods either have selection marker incorporated in the donor plasmid, which may lead to undesirable incorporation of the antibiotic resistance gene into the genome (Meier et al., 2010); or there is no antibiotic selection and much more clones need to be screened for insertion, which increases time, labor and material amount used. Thus, our study delivers a versatile, efficient, straightforward, and economic method to incorporate fluorescent tags in hiPSC.

This methodology is of particular interest when the aim is to characterize very delicate cell lineages such as neural cells. Once differentiated, these cells are very hard to genetically modify, therefore, CRISPR-editing of hiPSC followed by targeted differentiation offers a great tool to trace the expression of proteins of interest in real-time. One potential application is in developmental neurotoxicity testing (DNT), one of the most complex endpoints to address in regulatory toxicology (Bal-Price et al., 2018; Smirnova et al., 2018). Current *in vivo* approaches are prohibitively expensive, time-consuming, and have low predictivity for humans (Smirnova et al., 2014; Meigs et al., 2018). OLs migration, differentiation, and axonal myelination are key events of neural development but are very difficult to recapitulate *in vitro* using traditional monolayer cultures (Marangon et al., 2021; Hogberg and Smirnova, 2022). Therefore, the *PLP1* gene, an OL marker, was selected to be tagged with sfGFP to trace OL differentiation and migration during brain organoid maturation. It is also of high importance to design the tag to be a fusion protein, where PLP1 specific cellular location, as well as OPC and OL morphology, could be tracked over time. PLP1 fusion is also suitable for myelin quantification, as sfGFP protein is expected to be incorporated as a part of PLP1 protein into the myelin sheath. Specificity of the tag was demonstrated by: (a) a significant positive correlation of gene expression between *PLP1* and *GFP* throughout differentiation, (b) flow cytometry analysis showing co-expression of both proteins in 8-week bMPS, and (c) confocal imaging showing cellular colocalization between MBP and PLP1-sfGFP in mature oligodendrocytes at 8 and 10 weeks.

Expression of the *PLP1* gene during neural development is a dynamic process. Interestingly, we observed *PLP1-sfGFP* expression as early as in the NPC state, in line with previous reports demonstrating early *PLP1* expression in neural progenitors and its potential roles in neural development beyond myelination (Harlow et al., 2014; Guo et al., 2020). Based on morphology and MBP expression, we could differentiate immature OLs (GFP^+^/MPB^–^, 0-2 branches) from mature OLs (GFP^+^ MBP^+^, several long branches) in the bMPS. Branches of mature OLs were also wrapping around NF200^+^ axons. This provides an opportunity to develop algorithms for high-content imaging, which can distinguish cells based on morphology, so it is possible to identify which stage of maturation a given chemical or drug is affecting. In addition, the presence of an immature OL core and their migration before differentiation to mature OLs suggested the presence of OPC and OL cycling patterns (turnover, trans-differentiation of radial glia) in our model as previously observed in human CNS development and murine disease models (Richardson et al., 2006; Jakovcevski et al., 2009; Bradl and Lassmann, 2010). This makes our model suitable to study basic mechanisms of demyelinating diseases such as multiple sclerosis (MS) and potential therapeutics inducing remyelination.

To further confirm the proper functionality of the selected PLP1-sfGFP hiPSC clones, we monitored the expression of neural markers during neural differentiation. As expected, the differentiation followed the same trends as we observed previously (Pamies et al., 2017a; Chesnut et al., 2021; Modafferi et al., 2021; Huang et al., 2022): expression of OLs, astrocytes and neuronal markers was increased over time, while *NES*, a progenitor cells marker, decreased. In addition, we were able to observe interactions between OLs and mature neurons and trace PLP1-sfGFP alongside the axons, suggesting myelin formation in our fusion KI cell lines. Three-dimensional reconstruction of z-stacks at 8 weeks showed PLP1 localization around the axons and more strikingly, staining with MBP antibody at 10 weeks showed the tight arrangement of PLP1 and MBP proteins around the axons. Last, TEM images further confirmed the presence of wrapped myelin lamellae in our model as previously described by our group (Pamies et al., 2017a).

We also compared the differentiation of the PLP1-sfGFP line in two different neural media: B-27™ Plus Neuronal Culture System vs. B-27™ Electrophysiology kit. The use of Plus medium significantly improved gliogenesis in the course of neural development. According to the provider’s application note, Plus medium increases the survival of neuronal cells and enhances functional maturity. Interestingly, in our hands, we observed an enrichment of OL and astrocyte populations compared to Electro. This change made our model more physiologically relevant, closer to recapitulating the 1:1 neuron to glia ratio found in the human brain (von Bartheld et al., 2016).

Finally, to modulate the fluorescent tag, we challenged the bMPS with CPZ. Based on animal studies, we expected CPZ to decrease the PLP1-sfGFP signal by impairing myelination and promoting OL apoptosis (Hesse et al., 2010). To our surprise, we observed a significant increase in protein expression and overall intensity of PLP1-sfGFP assayed by flow cytometry. This may be explained by two reasons: (i) the absence of microglia in bMPS and (ii) OPC differentiation as a compensatory mechanism. Microglia is known to activate the inflammatory cascade to promote OL apoptosis and together with astrocytes clear myelin debris after demyelination (Skripuletz et al., 2013; Zhu et al., 2017), which is not occurring in our system. OPCs proliferation and migration to the sites of injury have been documented in response to the demyelination triggered by CPZ in murine models (Xing et al., 2014; Baxi et al., 2017). This could account, at least in part, for the increase in PLP1-sfGFP-signal we observed. In fact, quantification of the number of immature OLs (GFP^+^ MBP^–^) in the bMPS showed a significant increase after CPZ treatment. There was a decrease, however, in the number of mature OLs (PLP^+^/MBP^+^) and overall MBP expression in their processes after CPZ treatment suggesting demyelination. This observation confirms the importance of the incorporation of a fusion tag over a reporter tag and the use of subcellular imaging for analysis rather than solely quantification of the total fluorescence signal of a reporter in chemical and drug screening experiments. Importantly, this approach can only be effective when the fusion protein has followed functional validation in terms of subcellular localization, oligomerization, protein-protein interaction, and enzymatic activity (if applicable). The advantages of high-content imaging should be used to accelerate the screening of different chemicals/drugs and their effects on oligodendrogenesis and/or myelination.

Altogether, these experiments show the applicability of the model to study OPC and OL behavior in a human-relevant model. Our study (a) brings a very useful and cost-efficient methodology to incorporate fluorescent tags (KI) in hiPSC, so far, a very challenging and time-consuming procedure; (b) shows the optimal incorporation of the fusion fluorescent tag and normal functioning of the target cell type in our bMPS; and (c) demonstrates its applicability to study endpoints hard to model *in vitro* as well as its future potential to develop a multiplexed system for high-throughput drug and chemical screening.

## Data availability statement

The original contributions presented in this study are included in the article/Supplementary material, further inquiries can be directed to the corresponding author.

## Author contributions

LS conceived the study. JCR and LS designed the study and wrote the manuscript. JCR performed the majority of the research. CB and XC designed the CRISPR/Cas9 plasmids and provided insights. SC, YD, and YW provided the technical support. All authors contributed to the article and approved the submitted version.

## Footnotes

1
https://www.addgene.org/62988/

2
https://cctop.cos.uni-heidelberg.de:8043
