# Supplementary material for: Oligodendrogenesis and myelination tracing in a CRISPR/Cas9-engineered brain microphysiological system
Source: Front Cell Neurosci. 2023 Jan 19;16:1094291. doi: 10.3389/fncel.2022.1094291 (PMC9893511; doi:10.3389/fncel.2022.1094291)
Supplement: Supplementary file 1 [file Table_1.pdf]

Supplementary Table 1: List of primers used for template plasmid building, transfection efficiency and insert sequencing.

Section	Name	Sequence (5'→3')
Transfection efficiency	PLP Rvs	AGATGGGAGACGCAGCATTGTAGG
	PLP Fwd	AGGCCTGAGATAGTGTGGGTACAGCTATTC
	PLP Fwd set 2	TGCTGGAGGGAAGCTTCTGGGA
	PLP Rvs set 2	GCGCGGGTCTTGTAGGTGCCG
Insert sequencing	PLP Fwd set 2	TGCTGGAGGGAAGCTTCTGGGA
	PCR PCR REV 1	CGGAGCCTCCACCCTTTGCTATC
	PCR PCR FWD 2	CGAAACATGACTTGCTATAGCCTGG
	PCR PCR REV 2	GAAACCTCAGCAGTAGGGGCCAG
	PCR PCR FWD 3	TGCTAGATCAAACAGAGGTGTGGA
	PCR PCR REV 3	GGCCCATGAGTTTAAGGACGGCA
	PCR PCR FWD 4	AAGTTCATCTGCACCACCGGCAA
	PLP Rvs set 2	GCGCGGGTCTTGTAGGTGCCG
	PCR FWD 5	GAGAAGCGCGATCACATGGTCCT
	PLP Rvs	AGATGGGAGACGCAGCATTGTAGG
	PCR PCR FWD 6	AGGGGCCAAATATATTCTCTTTGGT
	PCR PCR REV 6	CCATCGGGGTCAGTGCTCTCTTT
	PLP rvs sequencing PCR	TCCATCGCCATCGGGGTCAGTGCT

Supplementary Table 2: List of primers used to sequence regions prone to off-target effects.

Gene	Name	Sequence (5'→3')
BSN	BSN Fwd	ATCATCGCCCCTCTGTGAAG
	BSN Rvs	AGGACCTGCCTGGAAAACTC
IFT52	IFT52 Fwd	CAGCAACTCCTACCCACTGA
	IFT52 Rvs	AAGCATGAGCCCATTGGAAGA
SPOCK1	SPOCK1 Fwd	CTGAGGGTGCACACCCGAGC
	SPOCK1 Rvs	AATCACCGGCAGTAACGGGGG
RP11-27G22.1	3rd off Fwd	CTGTCAATGGCAGAGACGCA
	3rd off Rvs	CAGCACCAACTGGTCTCCTT
AL591704.9	AL591704.9 Fwd	GGTAGAGGCGGTGCTGGGAATC
	AL591704.9 Rvs	CCCACCGTTCCAGCCCCATAG
C5orf34	C5orf34-Fwd	AGCACAAGTAAGGATCCTGGGCAA
	C5orf34-Rvs	AGGGAGTGTCTTGGGTAGCTTCTTT
GRM1	GRM1 Fwd	CCTGGCACCACCCACCATTACT
	GRM1 Rvs	CAAAGGCAGAAAGGCCAGTCAGGA
LINC00658	linc0068 Fwd	CCTATCTTCTGTGCCAGGGCTGC
	PLP OT5 Rvs	AACTCCGGGGTTGCACACAGTCA
RUNX2	2nd off Fwd	ATACTGGCTCAGCTGGTTTGA
	2nd off Rvs	ATGTCCAGTGGGCAACACAAG
AC073370.1	PLP OT7 Fwd	AAGGCCACGTCTGTAGCCAGC
	PLP OT7 Rvs	AAGCCCCACTCTCAGCACCAAC

Supplementary Table 3: List of TaqMan® Gene Expression Assay used for RT-PCR experiments.

Gene	Assay ID
18S	Hs99999901_s1
GFAP	Hs00909233_m1
MAP2	Hs00258900_m1
MBP	Hs00921945_m1
NES	Hs04187831_g1
PLP1	Hs00166914_m1
SYP	Hs00300531_m1
PDGFRA	Hs00998018_m1
CSPG4	Hs00361541_g1

Supplementary Table 4: List of antibodies used for immunofluorescence experiments.

Antigen	Host Species	Vendor	Catalog No.	Dilution used
β-Tubulin, Class III	Mouse	Sigma	T5076	1:1500
MBP	Rabbit	Cell Signaling	78896	1:200
PLP	Mouse	Bio-Rad	MCA839G	1:400
GFAP	Rabbit	Dako	Z0334	1:400
Neurofilament	Rabbit	Sigma	N4142.2ML	1:1000
PDGFRa	Rabbit	Cell Signaling	5241	1:400

Supplementary Table 5: List of antibodies used for flow cytometry experiments.

Antigen	Conjugation	Vendor	Catalog No.	Dilution used
β-Tubulin, Class III	AF647	BD Biosciences	560340	2uL/sample
PLP	APC	Milteny Biotec	130-120-275	2uL/sample
PLP	None	Bio-Rad	MCA839G	1uL/sample
GFAP	BV421	BD Biosciences	644710	1uL/sample
SOX2	PerCP-Cy5-5	BD Biosciences	561506	5uL/sample
NESTIN	AF647	BD Biosciences	560393	5uL/sample
TRA-1-60	PerCP-Cy5-5	BD Biosciences	561573	5uL/sample
NANOG	AF647	BD Biosciences	560279	5uL/sample
OCT3/4	AF488	BD Biosciences	560253	5uL/sample
