# Supplementary figures and images for: Oligodendrogenesis and myelination tracing in a CRISPR/Cas9-engineered brain microphysiological system
Source: Front Cell Neurosci. 2023 Jan 19;16:1094291. doi: 10.3389/fncel.2022.1094291 (PMC9893511; doi:10.3389/fncel.2022.1094291)

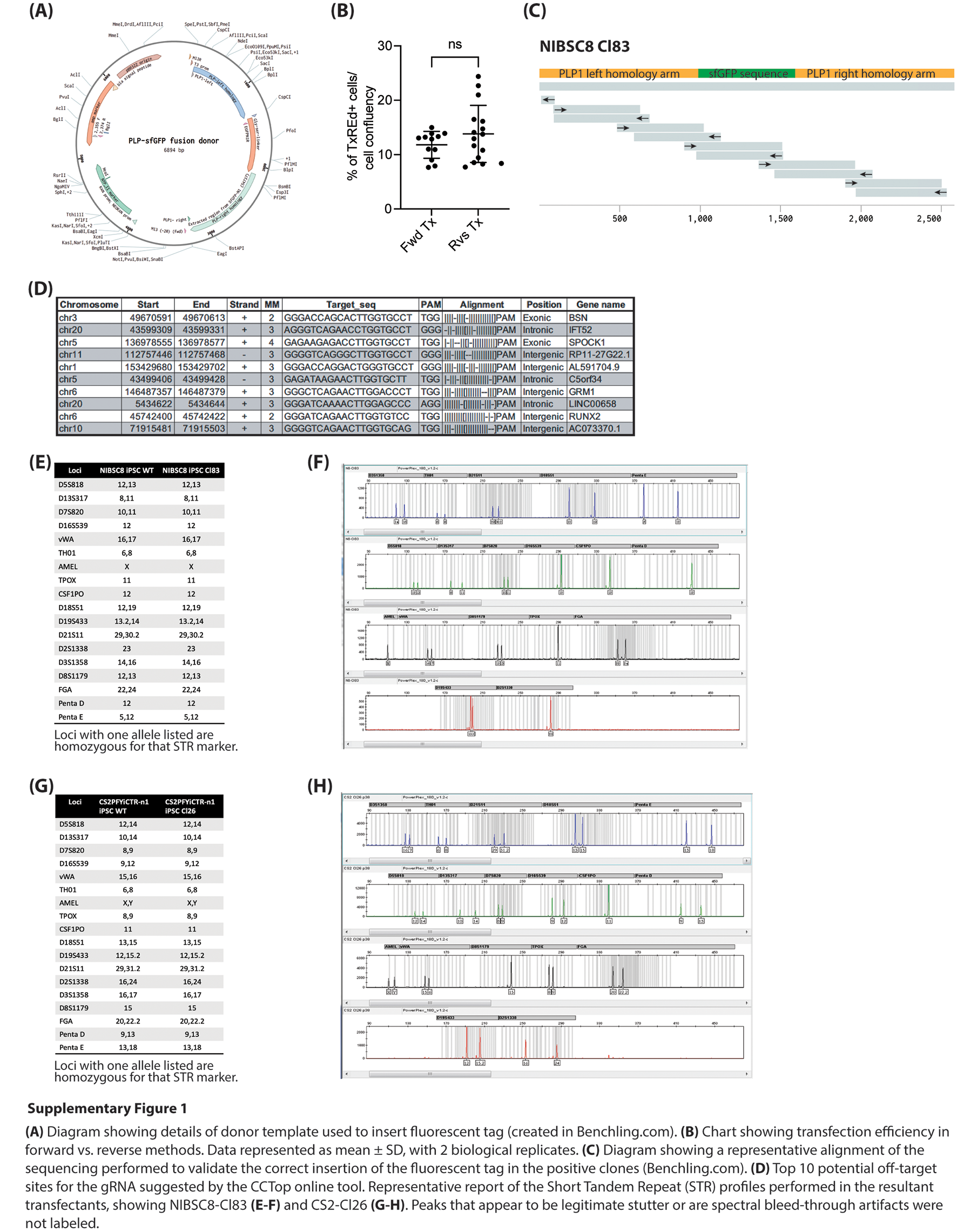

Supplement: Supplementary file 2 [file Image_1.tif]

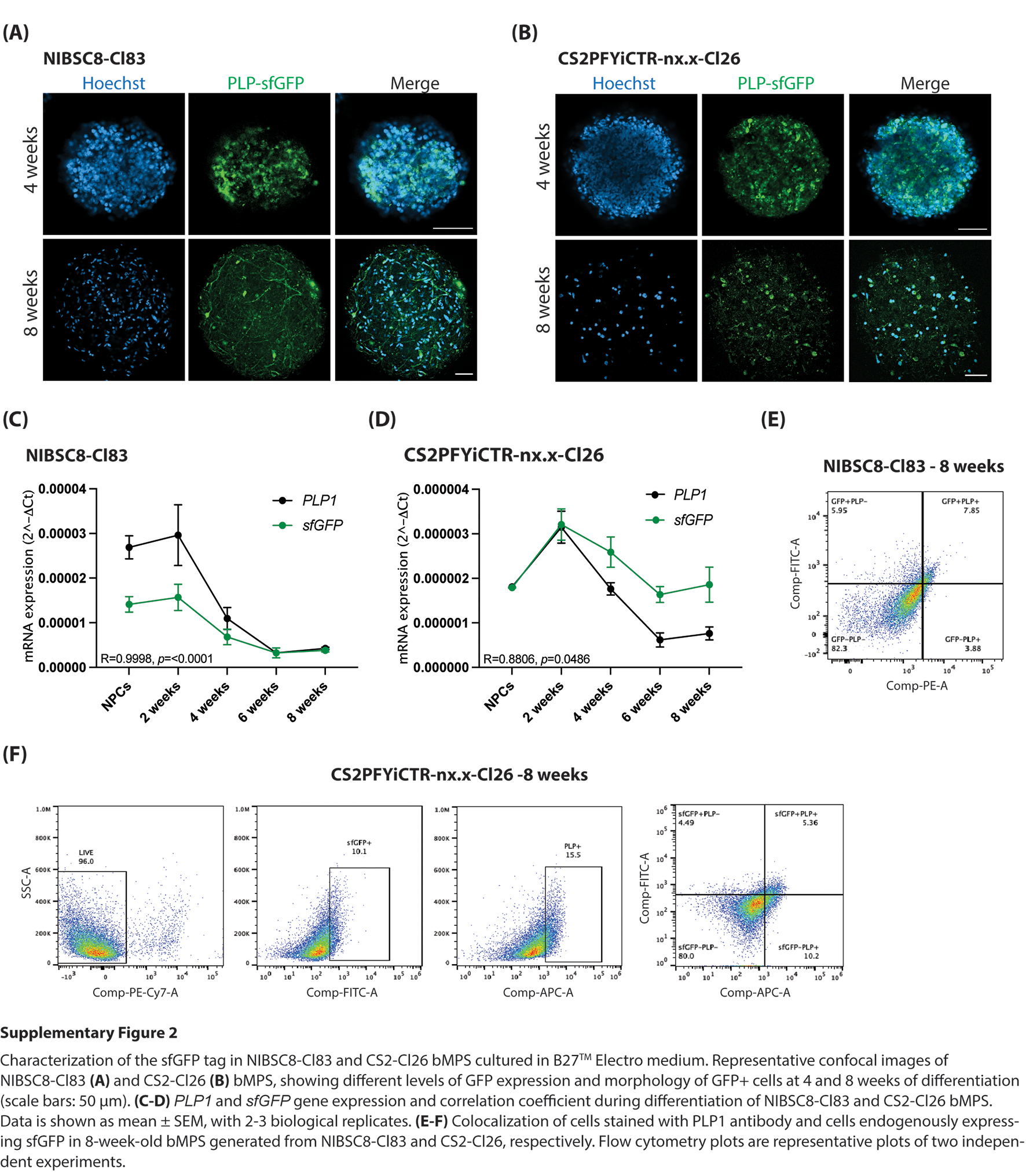

Supplement: Supplementary file 3 [file Image_2.tif]

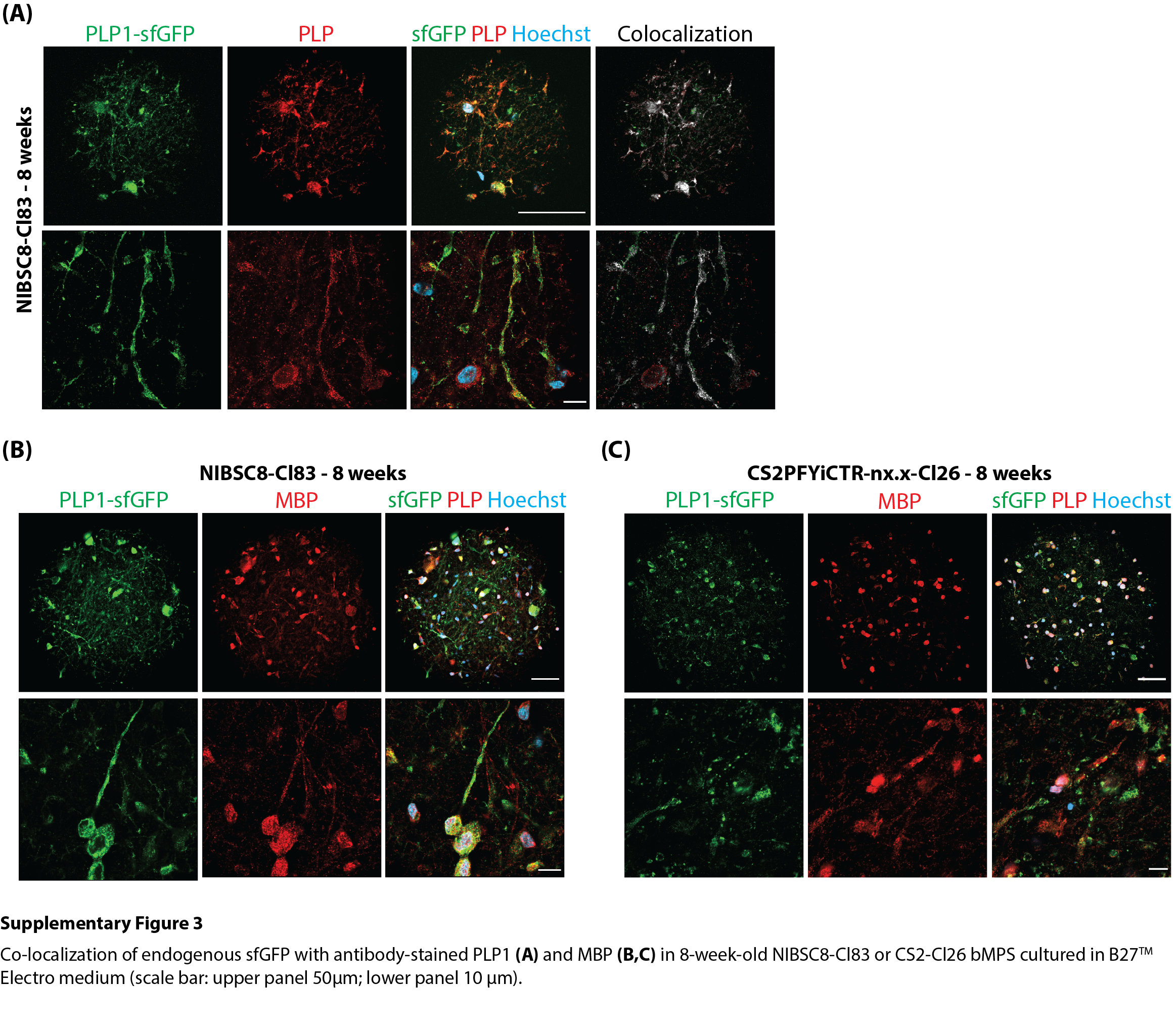

Supplement: Supplementary file 4 [file Image_3.png]

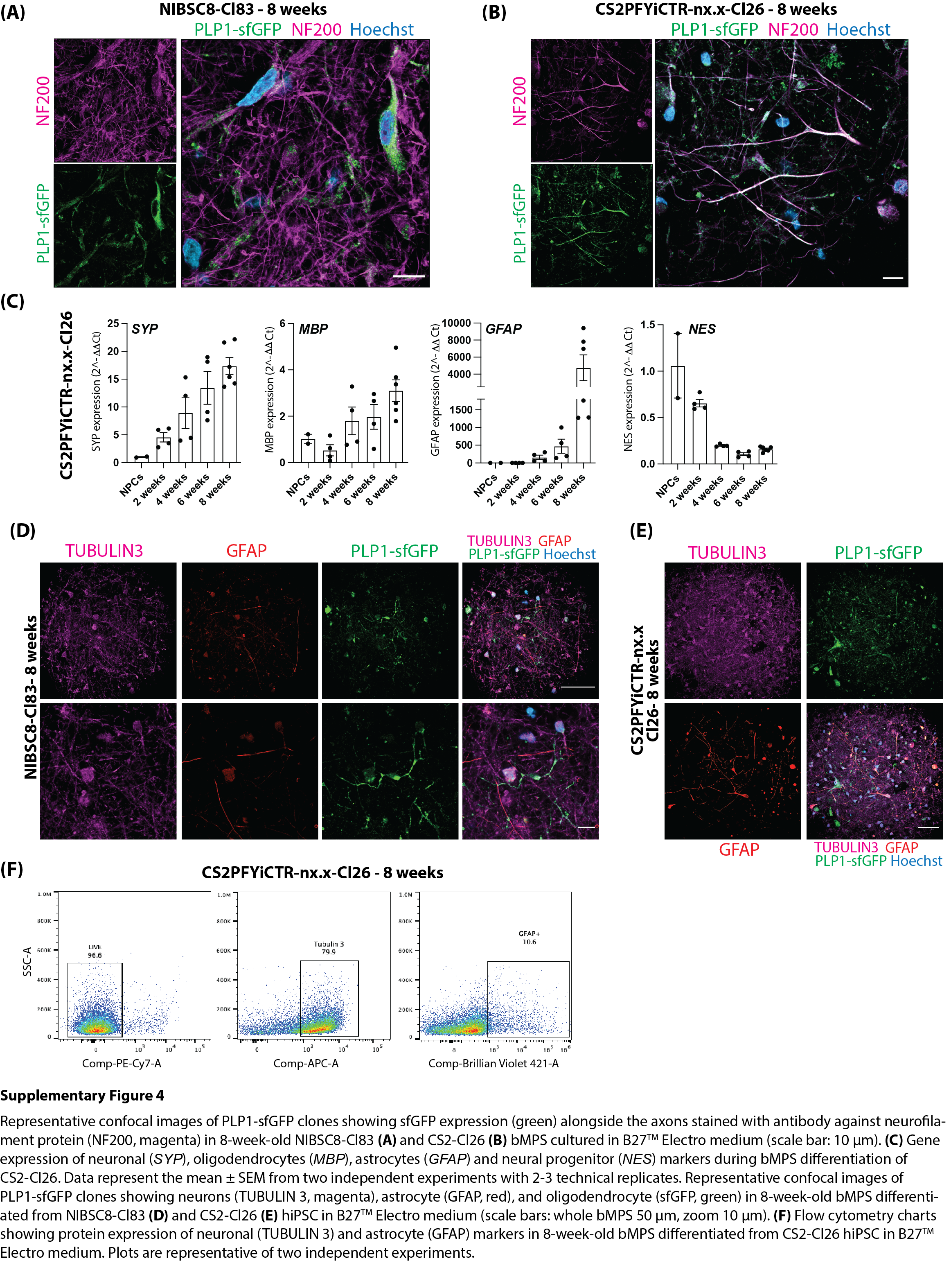

Supplement: Supplementary file 5 [file Image_4.png]

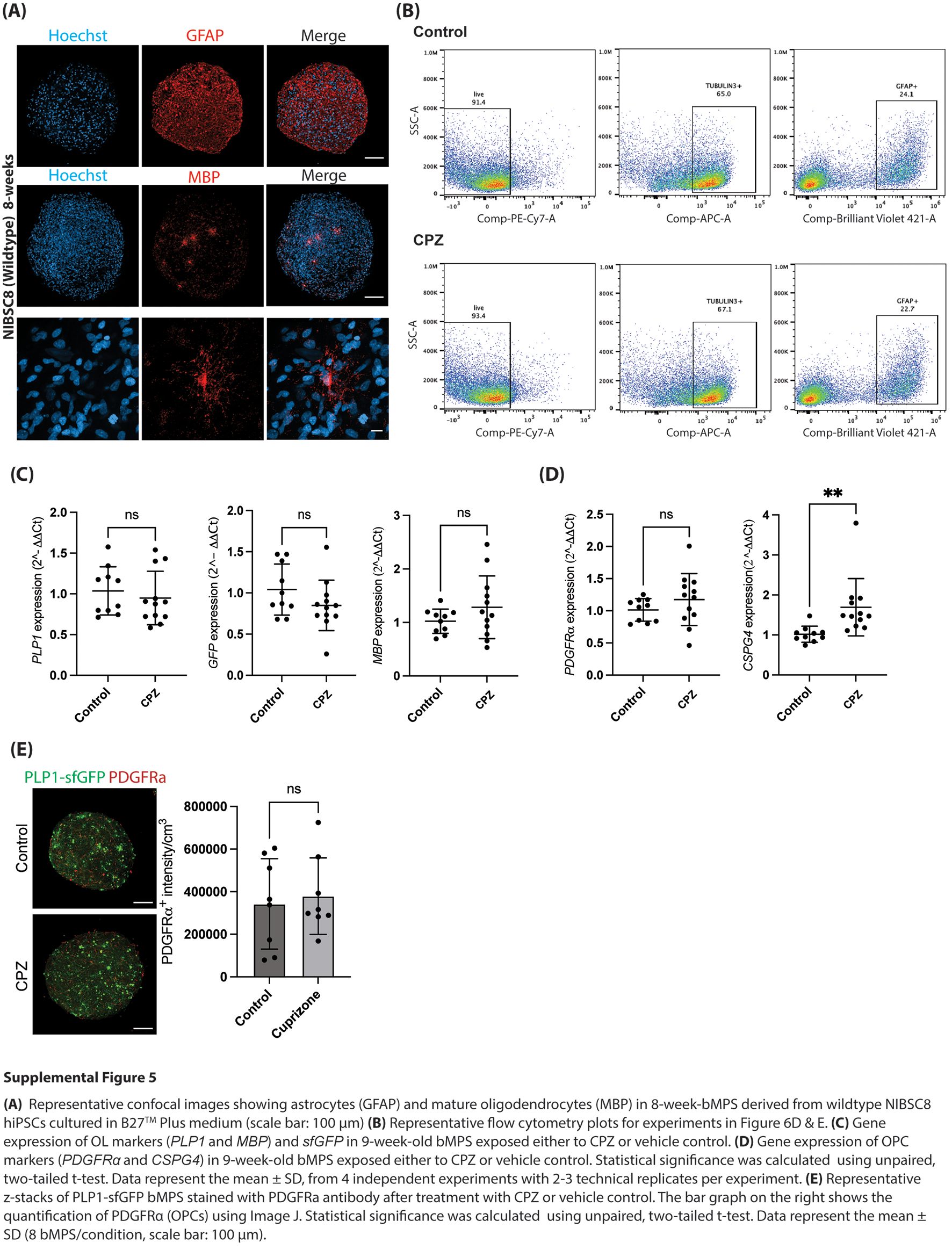

Supplement: Supplementary file 6 [file Image_5.tif]

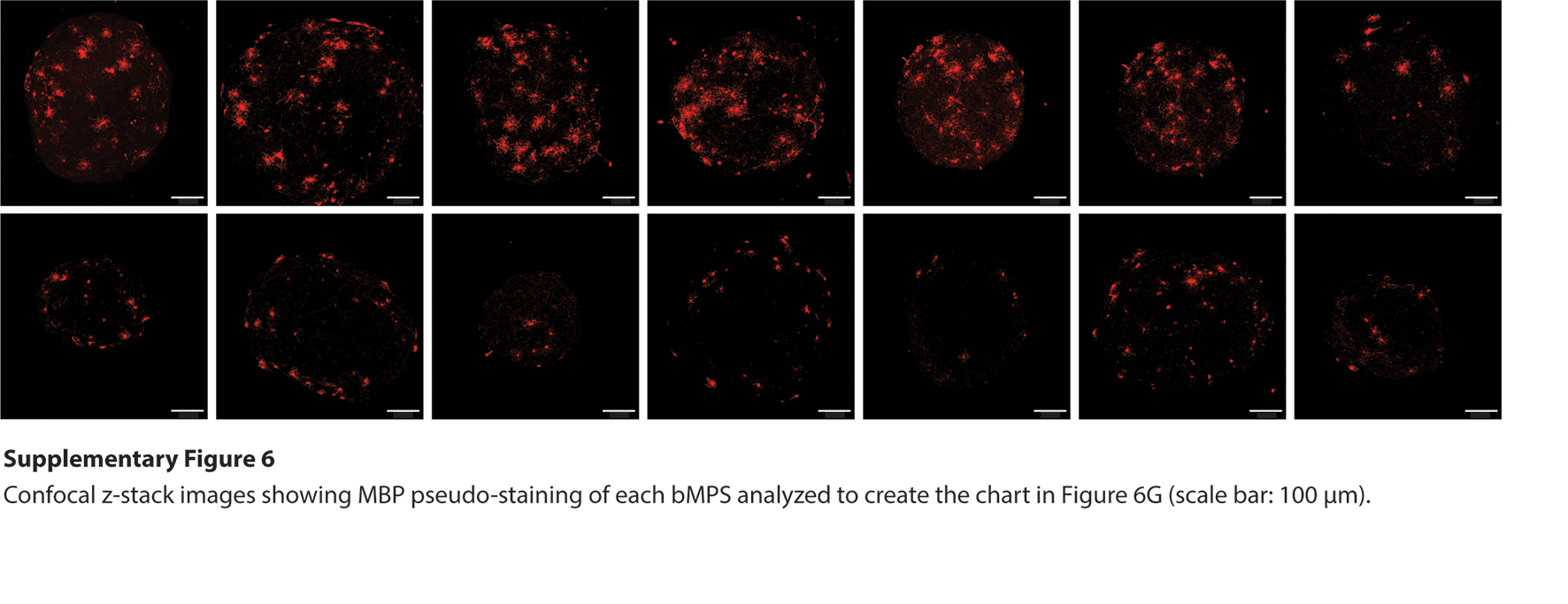

Supplement: Supplementary file 7 [file Image_6.tif]

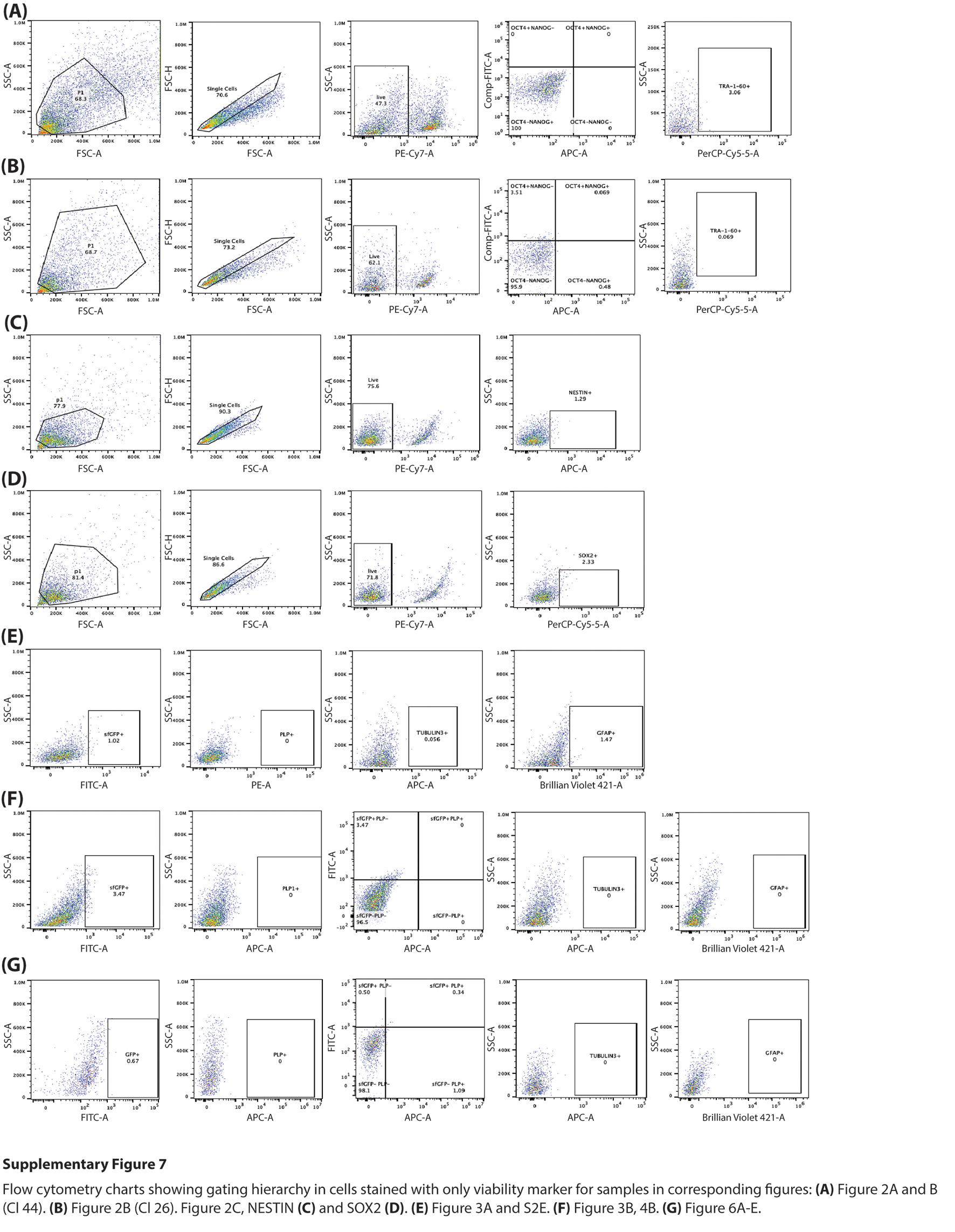

Supplement: Supplementary file 8 [file Image_7.tif]
